# Increasing the Frequency and Timeliness of Pain Assessment and Management in Long-Term Care: Knowledge Transfer and Sustained Implementation

**DOI:** 10.1155/2016/6493463

**Published:** 2016-03-03

**Authors:** Thomas Hadjistavropoulos, Jaime Williams, Sharon Kaasalainen, Paulette V. Hunter, Maryse L. Savoie, Abigail Wickson-Griffiths

**Affiliations:** ^1^Department of Psychology & Centre on Aging and Health, University of Regina, 3737 Wascana Parkway, Regina, SK, Canada S4S 0A2; ^2^Centre on Aging and Health, University of Regina, 3737 Wascana Parkway, Regina, SK, Canada S4S 0A2; ^3^School of Nursing, McMaster University, 1280 Main Street West, HSC 3N25F, Hamilton, ON, Canada L8S 4K1; ^4^Department of Psychology, St. Thomas More College, University of Saskatchewan, 1437 College Dr, Saskatoon, SK, Canada S7N 0W6; ^5^Strategic Affairs, Ste. Anne's Hospital, 305 Boulevard des Anciens-Combattants, Sainte-Anne-de-Bellevue, QC, Canada H9X 1Y9; ^6^Faculty of Nursing, University of Regina, 3737 Wascana Parkway, Regina, SK, Canada S4S 0A2

## Abstract

*Background*. Although feasible protocols for pain assessment and management in long-term care (LTC) have been developed, these have not been implemented on a large-scale basis.* Objective*. To implement a program of regular pain assessment in two LTC facilities, using implementation science principles, and to evaluate the process and success of doing so.* Methods*. The implementation protocol included a pain assessment workshop and the establishment of a nurse Pain Champion. Quality indicators were tracked before and after implementation. Focus groups and interviews with staff were also conducted.* Results*. The implementation effort was successful in increasing and regularizing pain assessments. This was sustained during the follow-up period. Staff members reported enthusiasm about the protocol at baseline and positive results following its implementation. Despite the success in increasing assessments, we did not identify changes in the percentages of patients reported as having moderate-to-severe pain.* Discussion*. It is our hope that our feasibility demonstration will encourage more facilities to improve their pain assessment/management practices.* Conclusions*. It is feasible to implement regular and systematic pain assessment in LTC. Future research should focus on ensuring effective clinical practices in response to assessment results, and determination of longer-term sustainability.

## 1. Introduction

Despite its high prevalence in long-term care (LTC; [1]), pain is often undertreated in such facilities, in part due to many patients' limited ability to communicate pain because of the cognitive and linguistic impairments that accompany dementia [2, 3]. Underassessed, uncontrolled pain is a major concern for LTC residents, not only impacting their quality of life [4], but also resulting in increased healthcare expenditures [5], as well as stress and burden among nursing staff [6]. Misattributing pain-related agitation or resident aggression [7] to other causes may further contribute to problems including the inappropriate prescription of psychotropic medications, which can have dangerous side effects and may hasten death [8–10].

Systematic pain assessment (see [11–14] for review) and pain management protocols have been found to be effective for residents with dementia. For example, regular pain assessments in LTC have been shown to improve pain management practices and reduce unnecessary polypharmacy, which is also beneficial for the work satisfaction of nursing staff (e.g., [7, 15–18]). In a large, randomized controlled trial of 18 LTC facilities, when residents were treated according to the pain management standards outlined by the American Geriatrics Society, as opposed treatment as usual, the treatment group experienced significantly reduced aggressive behaviours, neuropsychiatric symptoms, and pain, but not attenuated activities of daily living or cognition [19].

Despite the success of regular pain assessment, many facilities have not implemented these systematic protocols (e.g., [20]). Moreover, although proposals for systematic implementation research of regular pain assessment protocols have been put forward, empirical evaluations of feasibility have been extremely limited [21].

Public policy and clinical pain experts examined the possible reasons behind the insufficient implementation of systematic pain assessment and management protocols in LTC [22]. A group of policy and geriatric pain experts came to the conclusion that many pain assessment and management protocols are not being implemented because they fail to take into account fiscal and resource constraints of LTC facilities [22]. In response to this conclusion, they proposed a transformational protocol (with associated quality indicators) that took resource realities and feasibility into account [22]. The protocol was as follows: (1) all residents should be assessed using a valid and appropriate tool on admission (within 24 hours) and not less frequently than once a week; (2) residents who have a finding of moderate or greater pain (and nonself-reporting residents who have findings consistent with such levels of pain) will have a pain treatment plan implemented and documented within 24 hours; (3) within the 24-hour period following the pain treatment plan, pain and side effects of treatment should be reassessed and managed as appropriate; (4) in order to improve care for residents, pain assessment and treatment protocols become a component of ongoing quality improvement initiatives [22]. The protocol is consistent with authoritative clinical recommendations found in the literature [23]. It is also important to note that this protocol was formulated for use in a North American (Canada and USA) context. Although it is possible that the protocol would be suitable for other contexts, adaptations based on national standards/requirements may be necessary for adoption in other countries.

In a subsequent investigation [24], 167 Canadian frontline LTC clinical staff as well as administrators examined the protocol and concluded that it is both feasible and desirable. As a next step of the same program of research, we designed this implementation study. In doing so, we recognized that staff education can lead to increased knowledge about pain assessment, but changes in practice are often not maintained over time [25, 26]. For changes to be maintained, it has been suggested that management must reinforce the change through resource investment (including human resources) and ongoing support (e.g., [26, 27]). With such support, particularly the use of a Pain Champion, implementation is feasible [28]. As such, with full cooperation from management and staff, and with input from frontline personnel, we developed permanent implementation maintenance procedures that are congruent with empirically supported best practices for pain assessment. Management of participating facilities entered the implementation protocol with the intention of making it permanent and with the willingness to invest staffing resources to ensure its success.

Our approach was consistent with principles of implementation science outlined by Damschroder and colleagues [29] in their Consolidated Framework for Implementation Research (CFIR). Specifically, the CFIR was the result of review and analysis of 19 previous implementation models. The CFIR includes the following domains:

- *The Intervention*. This can be conceptualized as having “core” components (i.e., those that are essential) and the “adaptable periphery” ones (i.e., components that can be modified to the environment of the implementation). In our case, the intervention was the pain assessment protocol.
- *The Inner and Outer Settings*. These comprise the economic and sociopolitical contexts within which the organization exists and also the structural features of the organization itself. The inner and outer settings are highly linked and the distinction between them is not always clear. We carefully considered the context of our implementation through extensive discussions with facility administrators and through our preimplementation focus groups with staff. These considerations resulted in somewhat unique approaches to implementation/evaluation by each of the two facilities under study.
- *The Individuals Who Are Involved in the Implementation*. According to the CFIR, special attention should be paid to the individuals involved in the implementation process, as individuals both are carriers of organizational culture and also make choices that could have consequences for the implementation. In our case, we encouraged facility administrators to work collaboratively with staff in the implementation protocol and also facilitated staff input through our focus groups.
- *The Process of Accomplishing the Implementation*. The implementation itself needs to be an active change process and often needs to be championed by individuals within the inner or outer settings. There may be complexities and subprocesses involved in the implementation and these might not occur in a linear manner. Given this consideration, we encouraged the establishment of a Pain Champion within each participating facility who helped encourage and monitor the implementation process.

This project was conceptualized as a concurrent mixed methods design [30] occurring through a case series of two LTC facilities in two Canadian provinces. Specifically, we treated each of the facilities as a “case” and focused on their specific needs and structures in order to optimize implementation and evaluation of our evidence-based pain assessment protocol under real-world conditions. As such, quality indicators were tracked separately for each case. Mixed methods research is an increasingly common approach in implementation science and can be described as the systematic application of quantitative and qualitative research paradigms, generally in the service of responding to one set of research questions and hypotheses [30]. Since we were interested in responding to the unique aspects and needs of each facility, consistent with our case series design and in accordance with the framework proposed by Damschroder and colleagues [29], we allowed for some flexibility in the timing of implementation as well as in our evaluation approach in terms of the “adaptable periphery” (e.g., use of focus groups or interviewers based on the convenience and needs of each facility; allowing facilities to use their own strategies in facilitating pain assessments; relying on nursing staff judgement in choosing appropriate pain assessment and methods for their patient population) while retaining fidelity to the “core” components of our implementation protocol (e.g., delivery of a workshop on pain assessment and management; collection of quality indicators).

We expected that implementation of the pain protocol would result in significantly higher facility performance on a series of quality indicators. In addition, we used qualitative methods to clarify and better understand our findings; specifically, following the quantitative analyses, we derived a list of questions that we wanted to probe further and used qualitative analysis of focus group and interview data to answer these questions.

## 2. Method

**Figure 1:**
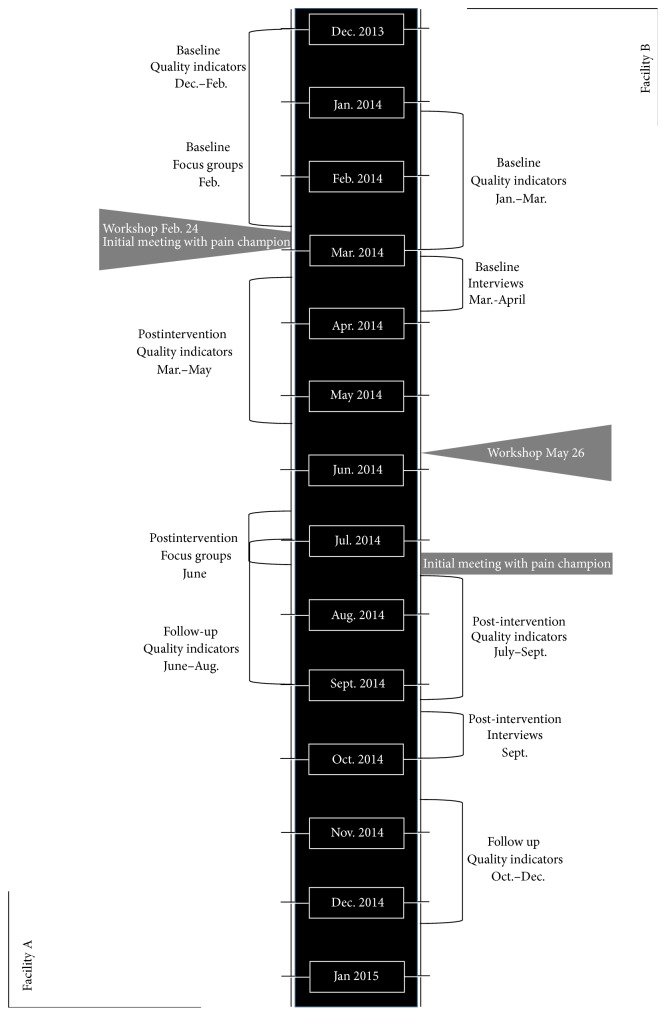
Timeline of implementation protocol.

As indicated above, our approach involved a case series design with two participating LTC facilities. For each participating facility, implementation of the clinical pain assessment protocol described above [22] was facilitated through a formal workshop (focusing on the protocol and on effective pain assessment methodologies), the use of a facility Pain Champion, and facility-specific strategies (e.g., electronic reminders about residents due for a pain assessment, scheduling assessments for different residents throughout the week). Evaluation of the strength of uptake occurred prior to and following the workshop/implementation protocol through both a visual examination of graphs summarizing quality indicators (consistent with case series methodology; in case series methodologies only large and obvious changes in data patterns, shown in graphs, are considered to be substantive [31]) and qualitative analysis of focus group and interview data. There were two evaluative components including a primary set of quantitative quality indicators assessed prior to the protocol (baseline), immediately after (post), and during a third, follow-up period (post; see Quality Indicators below). Further, qualitative research methods (focus groups and interviews) were used to complement these findings and these data were collected at two time points prior to (two months to one month prior to the workshop for Facility A and one month to two weeks prior to the protocol implementation for Facility B) and following the protocol implementation (approximately four months later for both Facility A and Facility B). A timeline of these procedures is presented in Figure 1.

### 2.1. Participating LTC Facilities

Our goal was to implement the pain protocol in two facilities from those associated with our network of research collaborators. We chose these facilities because they were quite different from one another (i.e., in two different provinces, one was hospital-based and the other was not) and on the basis of the administrative commitments to aim to sustain our pain protocols for the long term.

*Facility A*. The LTC facility is located at a midsized metropolitan area and provides 24-hour personal care to residents. Care is provided by registered nursing staff, continuing care aids (CCAs; also known as Personal Support Workers), and is guided by a staff physician. Person-centred values are apparent in the mission statement of the facility, as evidenced on their website where they describe maximizing resident quality of life, independence, dignity, and community participation. The facility has 127 beds and provides all levels of LTC. It is estimated that approximately 21% of residents in this facility have at least mild cognitive impairment, with 53% having moderate-to-severe dementia. Staff members at Facility A had been involved in prior research involving pain assessment and management protocols conducted by members of this research team. Our protocol was implemented throughout the entire facility and quality indicators (see below) are based on the population of the facility as a whole. 

*Facility B*. In Facility B implementation occurred in a single 33-bed unit within a larger 446-bed hospital dedicated to providing geriatric LTC and end of life care to older persons. Facility B provides care using a multidisciplinary team-led approach comprised of registered nurses, orderlies, physicians, occupational therapists, physical therapists, and social workers. In addition to LTC, the hospital provides respite care, as well as community support and mental health services. The organization is dedicated to providing high quality, personalized services to clients. Open communication, staff well-being, and commitment are among their other organizational values. The facility has a mandate to foster academic research and knowledge transfer, in an effort to bridge the gap between research and clinical practice. It is estimated that 30% of residents on this unit have symptoms of mild dementia with another 30% experiencing moderate-to-severe dementia.

### 2.2. Data Collected

*Quality Indicators*. Quality indicators are routinely monitored in healthcare to assist in the improvement of quality of care provision and to assess the performance of healthcare systems [32]. They assess the degree to which healthcare staff perform specific tasks to meet desired aims, such as the reduction of pain or agitation among residents [32, 33]. Consistent with the recommendations by Hadjistavropoulos et al. [22], quality indicators in this research included the percentage (%) of residents (a) with pain assessment documented within 24 hours of admission; (b) assessed for pain at least once per week; (c) with suspected pain and with pain treatment plan documented in the chart within 24 hours (documentation of any kind of treatment (e.g., administration of an analgesic medication, application of heat) in response to the pain assessment was considered to reflect a treatment plan); and (d) with suspected pain with reassessment within 24 hours to determine the effectiveness of treatment and side effects. Quality indicator information was provided by the facilities based on chart reviews. This information was collected by facility staff, under the direction of nurse managers within the facilities.

Facilities were instructed to utilize standardized pain assessment tools. However, the choice of specific tools was left to the discretion of the nursing staff of the facilities. As per facility standards, the Resident Assessment Instrument Minimum Data Set Version 2.0 (MDS; [34]) was generally used in addition to other measures. 

*Interview and Focus Group Data*. Textual data were collected from each facility through focus groups and individual interviews prior to (baseline) and following (post) the protocol. For Facility A, two focus groups were conducted at baseline, one consisting of 11 nurses and the other consisting of 9 CCAs. Following the implementation, two focus groups were also conducted, one consisting of 4 nurses and the other consisting of 10 CCAs. Due to the differing experiences, roles, and responsibilities of nurses and CCAs (as well as a power differential), we chose to separate these participants in accordance with focus group methodology that encourages homogeneity in the participants (e.g., [35]). For Facility B, six interviews were conducted at baseline, one with the facility's Clinical Nurse Specialist, one with the Director of Care, and four with nurses. The same participants from this facility were interviewed following the implementation. All participants provided written informed consent to participate in the focus groups or interviews.

In each instance, interviews and focus groups followed a semistructured format, involving an interview guide designed for this study (available on request from the authors). Questions related to gauging the problem of pain, the current state of pain assessment and management in each facility, and aspects of the protocol (i.e., baseline queries related to perceived challenges and postqueries related to determining aspects of the process that worked well and aspects that could be improved upon). Focus groups for Facility A were led by two members of the research team (S. K. and A. W.) and interviews for Facility B were led by a third member of the team (P. V. H.).

### 2.3. Procedure

*Workshop and Pain Champion*. A workshop, Pain Assessment among Persons with Dementia, comprised the key component of the implementation and occurred on February 24, 2014, for Facility A and May 26, 2014, for Facility B. These were conducted in person by a clinical pain assessment expert. All frontline staff were trained together in large groups of approximately 100. For Facility A, there were primarily people from the facility involved, with a few other people trained from outside also. Frontline staff trained in Facility A included nurses, CCAs, part-time, full-time, and night shift staff. For Facility B, this workshop was part of a more extensive education conference that also included people from other facilities. Most nursing staff members were trained including night staff but not all temporary staff. In addition to discussion of the implementation protocol, the content included a description of the problem of pain in LTC and the difficulties that seniors diagnosed with dementia have communicating. Empirically supported methods of pain assessment were described (e.g., self-report scales) as well as observational approaches to pain assessment with seniors who have dementia [36, 37]. Moreover, a clinical approach to assessment was presented and discussed [37]. Moreover, the need to integrate a wide variety of sources of clinical information was emphasized given the challenges in determining the extent of the intensity of pain manifestation based on observational tools alone.

During the workshop, the participants were also instructed in best practices for pain assessment. As indicated earlier, this protocol included a once weekly assessment of pain for all residents (or more often if pain was suspected) and documentation of a treatment plan within 24 hours at the latest. Reassessment of pain and assessment of treatment side effects were also recommended within 48 hours of treatment initiation. Written materials were provided to all workshop participants and a video discussing pain assessment methods and optimal frequency was also given to each facility to facilitate the training of any new staff or review by staff who had already participated in the workshop.

Managers were consulted and able to contact researchers as needed throughout the implementation process and following, via one-on-one telephone or in-person meetings. However, implementation proceeded smoothly without major issues and consultation was not necessary. In addition, for each facility, a nurse with a special interest in pain assessment and management was identified and invited to act as Pain Champion for the intervention. This person was responsible for the continued communication of workshop material to new staff members as well as assisting current LTC staff in managing complex resident pain situations. Additional release time was not provided for the Pain Champion. Facilities were fully supportive of participation in the project although additional resources were not provided. She also had access to the research team and clinical expert for any questions about protocol implementation. 

*Facility A Implementation*. Immediately following the workshop, the Director of Nursing met with the designated Pain Champion in order to discuss the best practice guidelines for pain assessment and management, and the steps needed to ensure implementation compliance on the units. The Pain Champion who was a Clinical Nurse Specialist, in addition to the duties described above, also ensured that pain was being assessed on a weekly basis and managed appropriately through regular in-person visits to the unit (i.e., daily Monday to Friday but not on weekends). In addition, an electronic charting system for pain assessment documentation was initiated, with electronic alerts (e.g., a different colour (pink) on screen until assessment for that day/shift was completed which then turned green). In addition, a monthly schedule of resident pain assessments was constructed which ensured that residents were evenly spread among the staff to prevent work overload. On the days when a resident was scheduled to be assessed, this system alerted staff members who completed the assessments. Moreover, electronic checks were conducted by the Pain Champion and followed up within 24 hours if staff missed documenting the assessment of a resident. The Pain Champion was available in this role throughout the course of the project and additional support was not necessary.

Through this scheduling process, the extra workload on any staff member was minimized since assessments were distributed across all staff (i.e., nurses and/or CCAs). If pain assessments were not completed, staff members responsible were followed up by the charge nurse. The facility's policy was updated, following the training workshop, to include nonpharmacological interventions (e.g., use of warm blankets, more frequent repositioning) and this was reviewed with nursing staff.

All quality indicators in this research (see Section 2.2) were assessed over three time periods: (1) a baseline period, which finished the week before the pain workshop; (2) a nine-week postimplementation period, which started two weeks after the pain workshop; (3) a nine-week follow-up period, which started four months after the workshop. Focus groups occurred at the same time at two time points, prior to and following the workshop. Details about timing of the evaluation processes are included in Figure 1. 

*Facility B Implementation*. Managers were consulted and were able to contact researchers as needed throughout the implementation process and following, via one-on-one telephone or in-person meetings. However, implementation proceeded smoothly and such consultation was not necessary. For this facility as indicated in Figure 1, there was a lag in time (due to summer holidays of key staff members) between the workshop and initiation of the implementation protocol. Approximately four weeks following the workshop, facility managers met with the Head Nurse on the unit in which the implementation occurred. The implementation protocol was reviewed along with strategies for incorporating these into care practices on the unit. Pain assessment was to be more frequent than previous and this posed a challenge for the staff, which they overcame by devising a formal schedule to ensure all residents were assessed on a weekly basis. The formal schedule enabled the Head Nurse to ensure adherence to the protocol. Once this schedule was in place, additional strategies were not necessary, as the protocol was adhered to and proceeded without incident. The Pain Champion, who was a Clinical Nurse Specialist, regularly visited the unit in person, ensuring that the protocol was followed appropriately and consulted with nursing staff about residents who were having more complex pain-related difficulties. Although the visits to the unit were not on a specific schedule, because the Pain Champion was the Clinical Nurse Specialist, she was on the unit several times a week during her work hours. When the Pain Champion was unavailable, the Head Nurse on the unit would act as a Pain Champion.

All quality indicators in this research (see Section 2.2) were assessed over three time periods: (1) an eight-week baseline period, which finished 12 weeks before the pain assessment workshop; (2) an eight-week postimplementation period, which started six weeks after the pain workshop (directly after the initial briefing of the Pain Champion); and (3) an eight-week follow-up period which started four months after the workshop. Interviews occurred prior to and following the workshop (see Figure 1).

## 3. Analysis

### 3.1. Quality Indicators

In accordance with our case series methodology, we used descriptive statistics and graphical plots to summarize the quality indicators for each facility [38].

### 3.2. Focus Groups and Interview Data

Focus group and interview data were analyzed using a basic descriptive approach (e.g., [39, 40]) with more of an emphasis on content rather than thematic analysis, although it has been noted that these approaches overlap substantially [41]. Analysis proceeded as follows. First, all transcripts were read in their entirety. Second, a first pass analysis parsed the text into broad “meaning units” (i.e., small units of text that conveyed meaning) that could be summarized in one or two sentences. Next, a list of queries specific to the implementation was derived. These queries were as follows. (1) At baseline, what aspects of the implementation did nursing staff believe would be the most challenging? (2) At baseline, what were staff members most eager to pursue or believed to be positive about the implementation protocol? (3) Following the implementation, what was positive about the process? (4) What could be improved upon? (5) What actions were nursing staff planning on carrying forward? In order to answer these queries a second-pass analysis occurred. Using the summary sentences from the first pass, relevant areas of text were identified. These areas of text were described more fully and examined across transcripts individually for each facility. Data were analyzed in the first instance by a primary researcher with extensive experience in qualitative analysis, in consultation with the other authors. The emerging structure and results of these analyses were checked and confirmed with two other members of the research team (also with extensive experience in qualitative analysis) who also conducted the focus groups and interviews.

## 4. Results

In accordance with the standard style of reporting case series research [42], results are described below per facility. We first present quantitative followed by qualitative results.

### 4.1. Quality Indicators

#### 4.1.1. Facility A

*(A) Percentage of Residents with Pain Assessment Documented within 24 Hours of Admission Using a Standardized Tool*. Facility A admitted 21 residents during the baseline period, 26 during the postimplementation period, and 21 during the follow-up period. Prior to the implementation, Facility A documented that on admission their residents were assessed 100% of the time for pain, albeit not necessarily through use of a standardized self-report or observational tool. After implementation, all residents were similarly assessed on admission with the exception of one week in which five residents were admitted but four were assessed for pain. At follow-up, all residents were assessed for pain on admission.

Prior to the implementation protocol, upon admission residents were assessed using the pain scale from the MDS Version 2.0 ([34]) in all instances. As part of the MDS, when residents reported having pain or were nonverbal at baseline, the Pain Assessment Checklist for Seniors with Limited Ability to Communicate (PACSLAC; [43]) was used once (4.8% of total assessments) and a 0–10 rating scale was used infrequently (5 times (23.8%) during three of the weeks). After the implementation, the MDS was similarly used for all newly admitted residents. The PACSLAC was used in one instance (3.8%) and a 0–10 rating scale (i.e., a standardized tool) was added in all but a few instances (24 times, 92% of residents assessed) demonstrating implementation success. That is, a 0–10 scale was not routinely used as part of the MDS. At follow-up, the MDS was used for all newly admitted residents. The PACSLAC was used in one instance (4.5%), and a 0–10 rating scale was used 20 times (91% of residents assessed).

**Figure 2:**
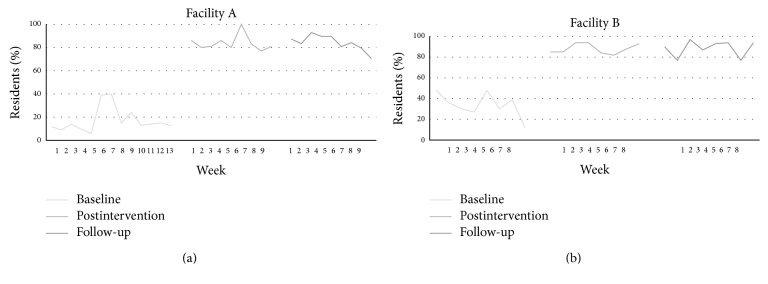
Percent of residents assessed each week with a standardized tool.* Note*. The percentages for Facility A are based on a total resident population of *N* = 121–127 (resident population varied slightly from week to week). The percentages for Facility B are based on a total resident population of *N* = 29–33 (resident population varied slightly from week to week).

*(B) Percentage of Residents Assessed At Least Once per Week Using a Standardized Tool*. During the baseline period, 17% of residents were assessed on average each week (SD = 10.62; mean *N* = 21, SD = 13.49). Following the implementation protocol, this percentage increased to 84% (SD = 6.74; mean *N* = 104, SD = 5.36), which was further maintained at follow-up (weekly mean = 84%, SD = 6.76; mean *N* = 105, SD = 8.70). Weekly details are presented in Figure 2.

Prior to the implementation, residents were assessed primarily with the MDS, which occurred 87 times during the 13-week baseline period. Also during this period, a 0–10 rating scale was used 21 times and the PACSLAC was used 241 times. During the postassessment period (9 weeks), the MDS was used 74 times, the 0–10 rating scale 327 times, and the PACSLAC 1061 times. During the 9-week follow-up period, the MDS was used 87 times, the 0–10 rating scale 280 times, and the PACSLAC 1031 times. 

**Figure 3:**
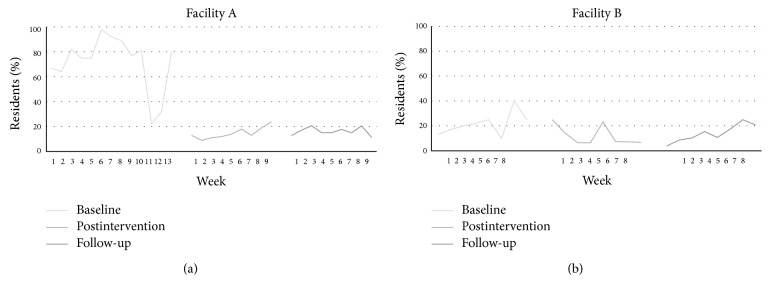
Percentage of residents who were found to have moderate-to-severe pain following the standardized weekly assessments.* Note*. For Facility A, the percentages are based on a total number of residents ranging from *N* = 8–50 prior to the intervention and *N* = 88–116 after the intervention and at follow-up (the denominator varied from week to week). For Facility B, the percentages are based on a total number of residents ranging from *N* = 4–16 prior to the intervention and *N* = 24–31 after the intervention and at follow-up (the denominator varied from week to week).

*(C) Percentage of Residents with Suspected Pain with Pain Treatment Plan Documented within 24 Hours*. Of those assessed for pain each week during the baseline period (i.e., of *N* = 21, SD = 13.49 residents who were assessed), on average 17 residents (SD = 14.46) (72%, SD = 22.06) had a finding of moderate-to-severe pain. Following implementation protocol, on average 15 residents (SD = 4.84) (15%, SD = 4.68) had this finding (i.e., of *N* = 104, SD = 5.36 residents who were assessed). At follow-up, on average 17 residents (SD = 3.86) (16%, SD = 3.19) were found to have moderate-to-severe pain (i.e., of *N* = 105, SD = 8.70 residents who were assessed). Weekly details of this quality indicator are presented in Figure 3. Each week at all three time points, 100% of residents with a finding of moderate-to-severe pain had a treatment plan initiated within 24 hours. 

**Figure 4:**
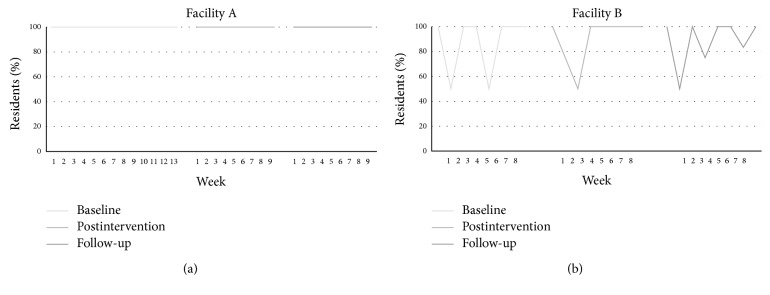
Percentage of residents assessed with standardized tool who had moderate-to-severe pain reassessed for pain within 24 hours.* Note*. For Facility A, the percentages are based on a number of residents ranging from *N* = 4–48 (the denominator varied from week to week). For Facility B, the percentages are based on a number of residents ranging from *N* = 1–7 (the denominator varied from week to week).

*(D) Percentage of Residents with Suspected Pain Reassessed within 24 Hours to Determine the Effectiveness of Treatment and Side Effects*. Each week at all three time points, 100% of residents with a finding of moderate-to-severe pain who had a treatment plan initiated within 24 hours were also reassessed for pain within 24 hours. Specific details about the reassessment were not documented at any time point, but, according to staff, tools were consistent with those used for admission and weekly assessments. See Figure 4 for weekly details. During the baseline period, no resident was specifically assessed for side effects within 24 hours after being treated for moderate-to-severe pain. During the postimplementation period, the same pattern was found with the exception of week one, in which two residents (15%) were reassessed for side effects. In contrast, during the follow-up period, on average 2 per week (SD = 2.30) (14%, SD = 15.46) who had a treatment plan initiated were assessed for side effects of treatment in the subsequent 24-hour period.

#### 4.1.2. Facility B

*(A) Percentage of Residents with Pain Assessment Documented within 24 Hours of Admission Using a Standardized Tool*. Facility B admitted three residents during the eight-week baseline period, one during the eight-week postimplementation period and two during the eight-week follow-up period. Prior to the implementation, Facility B documented that on admission their residents were assessed 100% of the time for pain. This was consistent after implementation as well and also during follow-up.

Upon admission during all three time points, residents were assessed by nursing staff first by asking the question “Do you have pain?” This question was asked of all verbal and nonverbal residents. If the resident said or otherwise indicated, “yes,” this was followed up with another measure. A follow-up measure was used in one instance (this occurred during the follow-up period when a 0–10 numeric rating scale was used). 

*(B) Percentage of Residents Assessed At Least Once per Week Using a Standardized Tool*. During the baseline period, on average approximately 11 residents (SD = 3.96) (34% on average, SD = 11.99) were assessed each week. Following the implementation protocol, this percentage increased to 88% (SD = 5.06) (mean *N* = 29, SD = 1.85), which was maintained at follow-up (weekly mean percent = 88%, SD = 7.64; mean *N* = 27, SD = 2.38). This demonstrates the success of the implementation. Weekly details are presented in Figure 2. For weekly pain assessments during the baseline period and also the postimplementation period, staff members first asked residents the question, “Do you have pain?” If the resident answered affirmatively, the staff followed up using the 0–10 rating scale. For resident unable to answer the question, the PACSLAC was used. Nonetheless, during the follow-up period, all residents were assessed either the 0–10 rating scale or the PACSLAC. 

*(C) Percentage of Residents with Suspected Pain with Pain Treatment Plan Documented within 24 Hours*. Of those assessed for pain each week during the baseline period, on average 2 residents (SD = 1.16) (21%, SD = 9.31) had a finding of moderate-to-severe pain. Following the implementation protocol, on average 3 residents (SD = 2.07) (12%, SD = 7.85) had this finding. At follow-up, on average 4 residents (SD = 1.83) (14%, SD = 6.90) were found to have moderate-to-severe pain. Weekly details of this quality indicator are presented in Figure 3.

During the baseline period, on average each week, 2 (SD = 0.99) (88%, SD = 23.15) residents with a finding of moderate-to-severe pain had treatment initiated within 24 hours of this finding. Similarly, following the implementation protocol, on average 3 (SD = 2.17) (91%, SD = 18.60) residents had treatment initiated. During the follow-up period, this weekly average was 3 residents (SD = 1.85) (89%, SD = 18.33). 

*(D) Percentage of Residents with Suspected Pain Reassessed within 24 Hours to Determine the Effectiveness of Treatment and Side Effects*. During the baseline period, on average each week, 81% (SD = 29.12) of those residents who had a treatment plan initiated were reassessed for pain (to determine whether the treatment was effective). During the postimplementation period, 58% (SD = 38.28) were reassessed and during the follow-up period, this average was 2 (65%, SD = 29.08). It is noted, however, that the actual resident numbers were small because very few had findings of pain and, consequently, a treatment plan. Weekly details are presented in Figure 4.

During the baseline period, on average each week 47% of residents (SD = 38.82), who had a treatment plan initiated, were assessed for side effects of treatment in the subsequent 24-hour period. During the postimplementation period, on average 73% (SD = 29.79) of residents who underwent pain management were assessed for side effects. Similarly, during the follow-up period, 52% (SD = 31.19) were assessed for side effects. Specific details about the tools used for reassessment were not documented at any time point but staff reported that the PACSLAC and/or 10-point scales were used.

### 4.2. Focus Group and Interview Data

#### 4.2.1. Baseline Transcripts

For Facility A, nurse participants believed they were already working positively towards assessing and managing pain in a way that was consistent with the implementation protocol. Similar to Facility B (see below), they noted that the protocol may be helpful in creating consistency among staff, as evidenced in the following exchange among nurse focus group participants:

*Interviewer: What other things do you think would be helpful?…*

*Participant: I think like a protocol, when there's pain this is what you do kind of thing you know. So there's consistency with every resident.*

*Participant: Just like a, what do you call that, algorithm.*

Participants from both Facility A focus groups identified insufficient time and staff resources to be potential barriers in the implementation process.

With regard to Facility B, during the baseline interviews, all participants stated that they believed the facility already did a good job assessing and managing pain and that the implementation protocol was consistent with their procedures. All participants were confident in the time and resources the facility had available to properly manage pain. They noted that the protocol may be more applicable in situations (i.e., different facilities) that were less advanced with treating resident pain. The Director of Care noted that the largest difference in the protocol was the frequency of assessing pain (i.e., weekly assessments, whereas the facility previously completed these monthly). Two participants stated that the protocol and quality indicators seemed to emphasize pharmaceutical interventions, rather than nonpharmaceutical treatments, the latter of which are emphasized to a greater extent in this facility. Two nurses believed the implementation would aid them in formalizing their existing process, potentially facilitating smoother communication among staff. One of these nurses stated:

*I really like this because this is pretty much what I have in my mind as far as having a protocol that everything together, to be able to follow through… follow up a lot better… it's quite good [current procedures] but there's nothing written, nothing specific so that we can all do the same thing on the unit.*

#### 4.2.2. Postimplementation Transcripts

For Facility A, as noted in the transcripts, the nurses were more involved with the implementation protocol than the CCAs. Although CCAs reported completing pain assessment documentation through formal reporting procedures, as a group they denied using formal assessment tools but rather relied on their knowledge of the residents and watching for behavioural changes. They did not report changes in practice (except for reporting procedures) due to the intervention. The nurses, on the other hand, noted that the implementation protocol resulted in quicker more frequent assessments, better communication with physicians, and greater use of nonpharmacological approaches to pain management. As stated by one nurse, “*I think it's good [the implementation protocol] because we become more alert, we become more diligent in pain assessment and intervention and the goal is for our residents to have more quality of life instead of just pain and that and crying*.” Formal reporting procedures and scheduling pain assessments were noted to be facilitative in terms of the implementation protocol. Both nurses and CCAs stated that communication with the other group to be a barrier to optimal implementation of protocol standards. Finally, resident denial of pain and lack of time were both noted to be barriers to pain assessment.

All participants interviewed from Facility B stated that the greatest change in practice was frequency of assessment. They reported now assessing weekly as opposed to monthly, which was their previous practice. This increased frequency was noted to be a potential barrier for continuing the protocol. For example, in their procedure for implementation, pain documentation occurred using the same system as other vital statistics, but the latter were only assessed on a monthly basis. In other words, there was discontinuity between the frequency of pain assessments and other assessments and a fear that this may result in less frequent pain assessments. Formal procedures and sheets for documenting pain assessment practices were reported to be facilitative to initiating and sustaining the protocol as was gathering baseline information about pain levels. Management support and the Pain Champion were reported to be crucial for initially making the changes in practice and also continuing these changes. All but one participant noted that the protocol resulted in positive changes (the former noting little change in practice). For instance, as reported by one nurse, the protocol helped

*to do a little bit more… that bit more with more confidence because we kind of… search a little bit where the pain comes from and adjust what we could give in terms of medicine… sometimes you know, like, we can't relieve it all completely, I think we can for some of those chronic pain but at least, you know, like to upgrade our protocol.*

## 5. Discussion

This research represents possibly the most important step in a program of research that began with the development of effective clinically useful approaches to assessing pain in nursing home residents with dementia (e.g., [36, 43]), continued with the demonstration that regular pain assessments lead to benefits for both residents and staff [8, 15], proceeded with the development of pain assessment/management guidelines designed to take resource realities into account [22], demonstrated that these guidelines are deemed important by frontline clinicians [24], and, in this investigation, examined the feasibility of actual implementation protocol in accordance with principles of implementation science literature [29]. This research further makes an important contribution to the literature, as research examining the feasibility of implementing such protocols is extremely limited [21].

This work responds to limitations in previous related studies, which found practice changes to be unsustainable over time due to lack of management support and resources [25, 26]. With our innovative use of a Pain Champion who was already employed in a leadership role within the facility, we address this limitation while still being able to respond to the fiscal realities of the LTC facilities (who were able to support the protocol without investing resources). Indeed, we demonstrated, through our research involving two different facilities in two cities, that regular pain assessments are able to be implemented in LTC without extra resources provided by the research team (other than a training workshop). It is our hope that this demonstration will encourage administrators of other LTC facilities to implement this or a similar protocol in order to increase the frequency of pain assessment and to improve the pain care of seniors, especially with dementia.

The importance of evidence-based training in empirically supported approaches is of central importance (a “core” aspect of our implementation protocol). In our case, this training was provided in the form of an interactive workshop. LTC facilities were able to implement the protocol and committed to aiming for long-term maintenance. Each facility tailored the implementation procedures to ensure that these were appropriate for the needs of the staff members completing the assessments and responding to resident pain-related needs. For example, one facility implemented an electronic scheduling system to facilitate timely and regular pain assessments. The other facility also implemented formal scheduling procedures for the same reason. CCAs were more involved in the implementation process in one facility (Facility B) than the other. It does not appear that this had an impact on the execution of the protocol but may have contributed to the difficulties reported by Facility A with regard to communication and cooperation between nurses and CCAs. We are of the opinion that CCAs have a role in the formal assessment of pain, given their often close relationships with residents, and indeed would encourage extensive involvement from this group of nursing staff. Optimal communication of assessment results and procedures with nursing staff is, nonetheless, essential since the nurses are ultimately responsible for care decisions and for any follow-up assessment that might be needed.

Although we found that pain was assessed more frequently following the implementation (indicating excellent adherence to the protocol), other quality indicators remained somewhat unchanged. It is important to stress, however, that the quality indicators were designed to assess protocol adherence and to be feasible for facilities to collect. They were not designed to assess effectiveness of pain management practices. That is, the facilities were following some of the best practices that were part of the implementation of the protocols even prior to its implementation. This may be due, at least partially, to the preexisting characteristics of the facilities in this study. These facilities, as compared to other facilities that were not as interested in pain assessment implementation work, likely committed to participate because pain management was on their agenda of important issues and recognized that uncontrolled pain has a major impact on resident quality of life and staff well-being. This is consistent with findings from the qualitative analysis; staff members from both facilities at baseline reported that some of the best-practice implementation procedures were consistent with their previous practices, although assessments with standardized tools became more frequent following protocol implementation.

Staff members in focus groups and interviews almost unanimously reported a positive impact of the assessment protocol that was implemented and anecdotally described numerous examples of how the protocol directly benefited the detection and treatment of undercontrolled pain among residents. Establishing each resident's baseline behaviours was viewed as necessary for assessing future pain. Focus group and interview participants reported that increased attention to pain assessment allowed for greater staff sensitivity to these issues and facilitated communication about pain-related issues, although in Facility A, insufficient communication between CCAs and nurses was noted as a barrier. Moreover, also as noted in the focus groups, our quality indicators were designed to evaluate protocol implementation and not pain management effectiveness. As such, they may not have been sensitive enough to the nuanced nonpharmacological interventions that the nursing staff engage in (e.g., frequent repositioning, warm blanket). Future research could focus more on these interventions, which may have produced a more robust effect in quality indicator outcomes.

No staff member reported that the residents became agitated by the frequency of assessment, although one staff member suggested that once a baseline was established, the frequency of assessment could be lessened. Future research is needed to determine whether there are differences in the effectiveness of pain management using a protocol such as the one recommended here as compared to routine care. Positive findings would encourage continuation of regular, at least weekly assessments. Moreover, it is possible that including pain assessment and management protocols into individual case planning research may be helpful in facilitating better care. For instance, once a baseline is established through weekly pain assessments, medically unstable residents may benefit from more frequent assessment whereas more stable residents may require less frequent assessment. Nevertheless, it is important that baseline is reestablished frequently, especially with residents who have limited ability to communicate verbally due to the presence of severe dementia. The protocol that we implemented incorporates individualization at least to some extent (e.g., although the recommendation is for a minimum of one assessment per week, the protocol also indicates that this frequency should be increased for residents with suspected pain). Nonetheless, future research could take into account more nuanced individualized case planning following a generalized procedure applicable to all residents.

From the perspective of the research team, it is clear that buy-in at all levels was a critical factor for successful implementation of this protocol, from management support, to the Pain Champion, and to the frontline nursing staff. Management support in particular has been shown to be critical for ensuring change [26, 27]. The establishment of a nursing position dedicated to pain assessment and management [28] is also important. Researchers dialogued regularly with facility managers, who were primarily responsible for ensuring that pain assessment best practices were being followed. However, it was the role of management and the Pain Champion to work with unit staff to personalize the procedures of protocol to ensure success. For instance, given the situation of the unit, management had to problem solve regarding the most practical and efficient method for ensuring that all residents were assessed on a weekly basis. This facility autonomy and ownership over protocol specifics are viewed as critical for successful maintenance of these procedures.

Facilities had some similar characteristics and also some differences that may have affected the results of this investigation. Staff of both facilities were enthusiastic about participating and Facility A had been involved in prior research regarding pain assessment. It is unclear whether this prior involvement may have impacted the results of our research. Moreover, Facility A provided LTC exclusively whereas Facility B was a unit in a larger hospital. These environments may have impacted the results of the research. Finally, Facility A had a larger resident population than Facility B which was a single unit in a larger hospital. The small number of residents in Facility B may limit the generalizability of our results. Nevertheless, both facilities showed a similar pattern in terms of increasing the frequency of pain assessments from baseline to postimplementation and follow-up.

The case series design is both a strength and limitation of this research. Case studies in general allow for an understanding of complex phenomena in a real-world setting [38]. Given the importance of individually tailoring the implementation protocol to characteristics of each facility (tailored by the facilities themselves), it is unlikely that alternate designs could have adequately allowed for this aspect of the research. Nevertheless, an approach examining specific aspects of the implementation protocol or without the flexibility we afforded the facilities in this investigation may lend itself to larger, controlled trials. For example, the timeline differed slightly for each facility in terms of the number of weeks of data which were collected and the spacing of research components. Moreover, each facility implemented additional strategies to aid them in executing the protocol, such as electronic monitoring. While a more stringent research approach might have had certain design advantages, we know that this would not have been feasible for the facilities within our national research network. Moreover, a more stringent approach would have likely not yielded agreement for participation. We also note that since this was a study of each facility's ability to implement our pain care protocol and not a study of patient outcomes, we did not report detailed demographic information for the patients. Similarly, we did not evaluate the nature of the treatment interventions (nor the appropriateness of assessment tool selection for specific patients) pursued by clinical staff. It would be important for future research to consider the extent to which demographic and diagnostic characteristics of the participants affect the implementation process. Moreover, future research could examine the quality of pain management interventions pursued by nursing staff in response to a pain assessment protocol such as the one that we implemented. Finally, the generalizability of our conclusions to LTC facilities with different characteristics (e.g., facilities based in remote or rural areas) from those of Facility A and Facility B remains to be investigated.

## 6. Conclusions

LTC staff were able to successfully implement and maintain a pain assessment protocol in two LTC facilities. That said, it would be important for future research to evaluate the sustainability of protocols such as ours over the longer term.

Protocol implementation success was dependent upon support from management, dedicated time of a Pain Champion for the protocol, and staff willingness to complete the protocol. The latter in particular was aided through facility-specific procedures designed to ensure staff were able to complete systematic pain assessments on each resident. It is unclear whether the increased frequency of pain assessments completed in this study resulted in better pain management but staff members reported specific examples when this occurred. Future emphasis on nonpharmaceutical interventions may create additional benefit to the quality of life of residents.

## Summary

Our program of research began with the development of clinically useful approaches to assessing pain in nursing home residents with moderate-to-severe dementia, continued with the demonstration that regular pain assessments lead to benefits, proceeded with the development of pain assessment/management guidelines designed to take resource realities into account, demonstrated that these guidelines are deemed important by frontline clinicians, and, in this investigation, examined the feasibility of implementation of a guideline-based protocol. Indeed, we demonstrated that regular pain assessments are implementable in long-term care without extra resources provided by the research team.
